# Heart rate variability helps classify phenotype in systemic sclerosis

**DOI:** 10.1038/s41598-024-60553-1

**Published:** 2024-05-15

**Authors:** Stéphane Delliaux, Abdou Khadir Sow, Anass Echcherki, Audrey Benyamine, Quentin Gomes de Pinho, Fabienne Brégeon, Brigitte Granel

**Affiliations:** 1 INSERM, INRAE, C2VN, Aix Marseille Univ, Marseille, France; 2 Explorations Fonctionnelles Respiratoires, AP-HM, Hôpital Nord, Marseille, France; 3 CNRS, CPT, Aix Marseille Univ, Marseille, France; 4 Laënnec Institute – Digital Sciences for Health, Aix Marseille Univ, Marseille, France; 5 Laboratoire de Physiologie, Cheikh Anta Diop University, Dakar, Senegal; 6 Service de Médecine Interne, AP-HM, Hôpital Nord, Marseille, France; 7 AP-HM, Microbes Evolution Phylogeny and Infections (MEPHI), IHU-Méditerranée Infection, Aix Marseille Univ, Marseille, France

**Keywords:** Systemic sclerosis, Heart rate variability, Nonlinear dynamics, Cardiovascular function, Machine learning, Machine learning, Neurophysiology, Nonlinear dynamics, Time series, Diagnostic markers, Autoimmune diseases

## Abstract

We aimed to develop a systemic sclerosis (SSc) subtypes classifier tool to be used at the patient’s bedside. We compared the heart rate variability (HRV) at rest (5-min) and in response to orthostatism (5-min) of patients (n = 58) having diffuse (n = 16, dcSSc) and limited (n = 38, lcSSc) cutaneous forms. The HRV was evaluated from the beat-to-beat RR intervals in time-, frequency-, and nonlinear-domains. The dcSSc group differed from the lcSSc group mainly by a higher heart rate (HR) and a lower HRV, in decubitus and orthostatism conditions. Stand-up maneuver lowered HR standard deviation (sd_HR), the major axis length of the fitted ellipse of Poincaré plot of RR intervals (SD2), and the correlation dimension (CorDim) in the dcSSc group while increased these HRV indexes in the lcSSc group (p = 0.004, p = 0.002, and p = 0.004, respectively). We identified the 5 most informative and discriminant HRV variables. We then compared 341 classifying models (1 to 5 variables combinations × 11 classifier algorithms) according to mean squared error, logloss, sensitivity, specificity, precision, accuracy, area under curve of the ROC-curves and F1-score. F1-score ranged from 0.823 for the best 1-variable model to a maximum of 0.947 for the 4-variables best model. Most specific and precise models included sd_HR, SD2, and CorDim. In conclusion, we provided high performance classifying models able to distinguish diffuse from limited cutaneous SSc subtypes easy to perform at the bedside from ECG recording. Models were based on 1 to 5 HRV indexes used as nonlinear markers of autonomic integrated influences on cardiac activity.

## Introduction

Systemic sclerosis (SSc) is a rare autoimmune disease combining vascular abnormalities with skin and deep organs fibrotic damaging that leads to the highest mortality among rheumatic diseases.

SSc is a heterogeneous disease and many efforts have been made for helping physicians to determinate patients’ prognosis. Since 1988, a sub-classification based on sclerosis skin extension assessed by palpation of 17 areas allows the diagnosis of the diffuse cutaneous SSc (dcSSc) and limited cutaneous SSc (lcSSc). Natural history, rate of progression, severity of organ damage and dysfunction, and mortality rate of disease depend in part on these 2 SSc subtypes. The dcSSc having the worst prognosis, identifying as soon as possible and with a robust manner SSc subtype remains critical for risk stratification and SSc patients care strategy^1^.

Clinical and biological evaluation has largely driven SSc risk stratification over the past decades, however, integration of new markers might improve disease phenotyping. As asymptomatic but also severe cardiac abnormalities, including myocarditis, congestive heart failure, arrhythmia, focal fibrosis, and impaired ventricular relaxation, are associated with systemic sclerosis and play a significant role in a considerable proportion of related fatalities^1^, markers of cardiac activity have been studied. Heart rate variability (HRV), that is often considered to reflect the autonomic nervous system (ANS) modulation of the sinus node rhythmic activity, has been proposed to assess cardiovascular function impairment in SSc since 1994^2^. Several studies have reported a lower spontaneous HRV in SSc patients compared to healthy controls in supine resting conditions^3,4^. The HRV index that is low-frequency spectral power has been associated with an enhanced resting sympathetic hyperactivity^5^. Additionally, SSc patients experienced first, a reduced heart rate (HR) response to tilt test highlighting impaired baroreflex modulation of the cardiovascular autonomic control and second a correlated high-frequency spectral power—lung function suggesting a respiratory sinus arrhythmia-supported HRV^5,6^. But HRV is also and more newly considered as the result of converging common and interacting sinus activity drivers that are largely interactive^7^. These drivers include, but non-exclusively, sympathetic and parasympathetic spontaneous and reflexively-driven tones, respiration pattern through frequency and volume activating pulmonary stretch reflex, non-vagal intrathoracic mechanical effects, hypercapnia, vagal-mediated arterial baroreflex effects, and local cardiac nodal (peptides) influences. Besides, SSc is known to potentially accumulate disturbances of several of these sinus node activity determinants such as autonomic nervous system, baroreflex function, lung function, and thoracic mechanics impairments. It seems therefore highly challenging to address the complexity of the issue in SSc patients through the only time- and frequency- domains.

Indeed, HRV has also been used to characterize cardiovascular function in children with systemic and localized scleroderma and no difference has been shown^8^. In adults of different SSc subsets, a recent study failed to prove the interest of some HRV indexes, particularly from spectral analysis, to distinguish dcSSc and lcSSc subtypes^9^. Nevertheless, HRV metrics and more specifically nonlinear ones as symbolic dynamics and conditional entropy confirmed their ability to detect a predominant sympathetic modulation of sinus node rhythmic activity at rest and a blunted response to orthostatism stress especially at the SSc early stage^9^.

All these exploratory works highlight the interest of HRV analysis, particularly through nonlinear metrics, to study SSc impact on cardiovascular function. To date, none provide discriminant HRV markers to distinguish the different SSc subtypes. A bioinformatic marker for SSc subtypes classification is still lacking. The aim of this research was to propose a classifying tool including HRV indexes only, easy to compute, and to be used at the patient’s bedside. To do so, we tested the hypothesis that a combination of few variables describing sinus node rhythmic activity and particularly describing the nonlinear properties of its dynamics as correlation dimension (CorDim), could allow to distinguish dcSSc from lcSSc. In accordance with our knowledge and experience previously acquired^10^ and because CorDim is typically used as an index of the overall complexity of the system dynamics estimated from a time series, CorDim should be specifically efficient in multiple physiological functions interactions situations such as that observed in SSc patients performing a stand-up test.

To meet this challenge, we compared in dcSSc and lcSSc patients HRV at rest and in response to orthostatism. We computed several linear and nonlinear HRV metrics from time-, frequency-, and nonlinear- domains as a step of a data engineering process. Several classifying models (metrics combinations) and classifying methods (classifier algorithms) have been compared to help the practitioner to choose the best classifying model according to the clinical needs.

## Methods

### Study design and participants

We did a retrospective observational study of 58 consecutive patients aged 18–74 years and suffering from SSc. Patients were identified from the consulting population of the internal medicine department of north hospital, Marseille, France, from 2018 to 2021. Power analysis was performed to determine sample size. To achieve a power of 80% (type II error) and a level of significance of 5% (two-sided type I error) for detecting a 50% group difference (i.e. > 2 vs. < 1 degrees of freedom in the RRI generating dynamical system) in expected mean value (2.2 ± 0.8, based on previous study^10^ and preliminary data) of the correlation dimension (CorDim), 16 patients (8 per group) were necessary. To ensure high models performances we included more than necessary subjects. Moreover, a posteriori calculation of Cohen’s d metrics (equal to 1.16761) as well as Hedges’ g metrics (equal to 1.455829) that is usually used for unbalanced groups showed that size effect was very strong in our study and that sample size = 76 (16/60) was sufficient.

Inclusion criteria were 18–80 years of age, diagnosis of SSc confirmed by an internist medical doctor according to the 2013 classification criteria for SSc^11^. No specific criteria on the presence or absence of dysautonomia was checked at this step. Exclusion criteria were known heart disease, beta-blockers medications, and diabetes mellitus. Neither heart disease nor diabetes has been detected. 3 patients were identified as being treated by beta-blockers for systemic hypertension and were therefore not included. We included 60 patients and 2 were not analyzed because of incomplete data collection.

Informed consent was obtained from all subjects by the institution concerning care data exploitation for research purposes. The protocol of data exploitation was developed in accordance with the declaration of Helsinki and health data analysis approval has been provided on January 2020 by the Assistance Publique–Hôpitaux de Marseille Data Protection Officer and local ethics committee under the references PADS19-352 and WZTVF7.

### Procedures: clinical and paraclinical features

New or already known patients referred for SSc to the internal medicine department from 2018 to 2021 were retrospectively screened. All patients included in our study fulfilled the 2013 American college of Rheumatology/European league against rheumatism classification criteria for SSc^11^. According to the internist medical doctor specifications, patients had been explored through paraclinical biological, imaging, and functional tests including when necessary: autoantibodies tests, chest CT scan, echocardiography, right heart catheterism, lung functional tests, esophageal manometry, and cardiovascular dysautonomia checking through spectral heart rate variability assessment. The heart rate variability recordings were made prospectively on medical justifications for routine care while data were retrospectively re-analyzed with complementary technics for our research purposes. SSc patients were classified into dcSSc or lcSSc subtypes by the internist medical doctor expertise according to the 1988 LeRoy et al. classification^12^. Accordingly, to the extent of skin sclerosis, two groups were considered, dcSSc and lcSSc groups.

### Heart rate variability study

The subjects were instructed to refrain from exercising, drinking alcohol, coffee or tea, and sleep deprivation for at least 24 h before their participation. The day of the HRV study, room experiment was quiet and its temperature kept constant. Subjects were asked to lie down in the examination bed and to relax with free breathing. Three lead electrocardiogram (ECG) was acquired (ECG100C, UIM100C, and MP150A-CE Biopac equipment, BIOPAC Systems Inc., Santa Barbara, CA, USA) during 15 min in supine resting condition and then during 10 min in orthostatic resting condition after stand-up. The data were digitized and stored on a personal computer for further analysis. Two periods of 5 min were extracted and analyzed: (1) 5 min of supine rest (Decubitus) after at least 5 min of stable ECG signal, and (2) 5 min of orthostatic rest (Orthostatism) starting immediately after stand-up phase (< 10 s) noise stabilization. Signals were conditioned and processed as follow and previously described^10^. Raw signals were sampled at 1000 Hz and digitized with a 24-bit analog-to-digital converter. Non-evident non-stationarity, such as very slow drifting of the mean or sudden changes of the variance, was observed after 3-order polynomial detrending. No additional filtering technique such as integration was used. Pan and Tompkins real-time QRS detection algorithm was used to automatically detect R-waves and build the RRI series. Tachograms were then firstly visually checked and corrected for artifacts (including ectopic beat management, i.e., detection, cancelation, and interpolation) when necessary. Then, all cardiovascular data analysis was performed from the 5 min beat-to-beat time series constituted from inter-beat intervals from ECG R waves, i.e., the RR intervals (RRI) that were extracted from raw signals. Discrete original RRI series were resampled by cubic spline interpolation with a 4 Hz sampling rate to generate equidistantly sampled time series x(i), i = 1, 2, …, N. The HRV was assessed according to the consensual standards^13^, using Kubios software (Kubios HRV, 2.1, Biosignal Analysis and Medical Imaging Group, Kuopio, Finland), computing HRV metrics in Decubitus (_S1) as well as in Orthostatism (_S2). We also assessed stand-up induced HRV changes (_delta) calculating the difference between orthostatic and decubitus values for each variable.

### Time-domain analysis of HRV

Several classical metrics were used as indexes of the total variability that arises from both random and periodic sources^14^, including: the mean RRI (mean_RR) and mean HR (mean_HR), the standard deviation of the RRI (sd_RR) and of the HR (sd_HR), the square root of the mean squared differences between adjacent normal RRI (RMSSD), the count of successive normal beat lengths that differed more than 50 ms (NN50), and the percentage of successive normal beat lengths that differed more than 50 ms (pNN50).

### Frequency-domain analysis of HRV

Different metrics were used to characterize the periodic oscillations of the studied time series using the estimated power spectrum by fast Fourier transform Welch’s periodogram technique. These metrics included the centroid frequency (expressed in Hz) and power (_power, expressed in ms^2^) in 2 frequency bands of interest: high frequencies (HF, 0.15–0.4 Hz) and low frequencies (LF, 0.04–0.15 Hz). The total power (tot_power) and LH_power/HF_power ratio (LF_HF_power) were also computed. The HF_power and LF_power were also expressed as the percentage of tot_power (HF_power_perc and LF_power_perc, respectively) and normalized units (HF_power_nu and LF_power_nu, respectively). These variables were said^13–15^ to be indexes of the total variability of the heart rate (tot_power), the modulation of the sinus node activity by the parasympathetic component of the ANS (HF_power_nu), the modulation of the sinus node activity by both the parasympathetic and sympathetic components of the ANS (LF_power_nu), the influence of the temperature, and the sympathetic/parasympathetic balance (LF_HF_power).

### Nonlinear-domain analysis of HRV

A set of metrics are known to catch and highlight non-linear properties of the studied time series. We plotted the RRI of rank n + 1 as a function of the RRI of rank n (lag 1 Poincaré plot) and calculated its SD1, SD2 and SD1/SD2 defined as the standard deviation of the instantaneous beat-to-beat RRI variability (minor axis of the fitted ellipse), the standard deviation of the continuous long-term RRI variability (major axis of the fitted ellipse) and the axis ratio, respectively^16^. They were respectively used as non-linear indexes of (1) the rapid changes in the RRI and thus of the parasympathetic sinus node control^17^, (2) the effects of both parasympathetic and sympathetic components on the sinus node activity^18^, and (3) the relationship between these components, which is the ratio of the short interval variation to the long interval variation. We also used other non-linear tools to characterize the RRI dynamics, as previously described^10^. Recurrence plot analysis quantification (minimal line length (Lmin), mean line length (Lmean), maximal line length (Lmax), divergence (DIV), recurrence rate (REC), determinism (DET) and Shannon entropy (ShanEn)) was performed with embedding dimension, lag, and threshold distance set to m = 10, τ = 1, and $r= \sqrt{mSD}$ of the RRI time series analyzed, respectively. We computed detrended fluctuation analysis α1 and α2 coefficients (alpha1 and alpha2, respectively) with segment length set to n ∈ (4,16) and n ∈ (16,64), respectively. Approximate Entropy (ApEn) and Sample Entropy (SampEn) estimates of each RRI time series were computed with m (embedding dimension) and r (filtering level) set to 2 and 0.2 SD of the RRI time series analyzed, respectively. Finally, we also estimated correlation dimension (CorDim) of the RRI times series^19^. Indeed, first, non-linear deterministic measures of heartbeats better detect the autonomic changes related to mortality than the stochastic indexes; and second, what the time-dependent non-linear metrics show as indicators of cardiac vulnerability to lethal arrhythmias are transient non-stationary shifts of dimension of the heartbeat dynamics to a low value^20^. CorDim quantified the time self-similarity of a signal, i.e., of the RRI time series. CorDim can be considered as an estimation of the lower threshold of the number of degrees of freedom for the underlying system generating the observed data and is typically used as an index of the overall complexity of the system dynamics estimated from a time series^21^. CorDim was computed as described by Grassberger and Procaccia^22^ with r = 15% from the standard deviation of the RRI. Accordingly, the first step is to construct the correlation integral (N,m,r) function. The correlation integral counts the fraction of pairs (Xi, Xj) whose distance is smaller than r and is defined as:

$$C\left(N,m,r\right)=\frac{1}{N\left(N-1\right)} {\sum }_{i=1}^{N}{\sum }_{j=i+1}^{N}H\left(r-\left|xi-xj\right|\right)$$

where Xi and Xj represent phase-space trajectory points, N represents the total amount of phase-space points, and H represents the Heaviside step function, i.e., H(α) = 0 if α < 0 and H(α) = 1 if α ≥ 0. D2 is subsequently computed as:

$$CorDim=\underset{r\to 0}{lim}\frac{log(C)}{log(r)}$$

### Outcomes

The main outcome was the ability to express the SSc subtype as a function of HRV, i.e., to classify the SSc clinical form as diffuse or limited of a patient using heart rate dynamics metrics as markers. Secondary outcomes were the link between HRV metrics and SSc skin extent, and between HRV metrics and lung function.

### Statistical analysis

All statistical computations were performed with Python 3.10 LTS.

#### Descriptive statistics

The quantitative variables were first tested for normality distribution with the Shapiro–Wilk normality test. Accordingly, means or medians groups’ differences (i.e., dcSSc vs lcSSc) were tested performing independent t-tests, paired t-tests, or Wilcoxon-Mann–Whitney tests when necessary for continuous variables, and Wilcoxon-Mann–Whitney or Kruskal–Wallis tests for ordinal variables. The qualitative categorical variables were assessed through modalities headcounts and groups’ differences were tested performing Chi-square or Fisher exact tests. Linear correlations between quantitative variables were estimated computing Pearson's and Spearman’s correlation coefficients when necessary and hypothesis of correlation was tested performing a t-test. A p-value < 0.05 was considered as statistically significant, nevertheless all variables having an unadjusted univariate p-value < 0.1 (n = 33) were considered for feature engineering. Furthermore, specifically for HRV analysis, and with a dual objective of simplifying the interpretation, particularly in terms of physiological outcomes, and limiting the expansion of type I error, a two-way repeated measures ANOVA analysis with Bonferroni correction was employed. The first factor considered was the clinical form (dcSSc or lcSSc), while the second factor was the position (decubitus or orthostatism). Non-parametric ANOVA (Kruskal–Wallis) was conducted when necessary. For secondary outcomes, simple and multiple linear regressions were performed to predict quantitative variables while simple and multiple logistic regressions were performed to predict bimodal categorical variables. Ordinary Least Squares optimizing method were used in both cases. Significance was set to p-value < 0.05.

#### Features engineering

To limit effects of collinearity on features extraction procedure, we identified all pairs of variables being highly correlated (R^2^ > 0.9) and, according to their physiological explainability, excluded the less appropriate variables (n = 9). Quantitative data were then normalized (min–max scaling) for subsequent steps. On one hand, five features selection algorithms (Random Forest, Extra Tree Classifier, Select K Best, Generic Univariate Selection, and Recursive Feature Elimination) were used to rank the 24 selected HRV variables. Ranking was performed on variable importance (explained variance) through a homemade redundancy weighted score taking into account, for each variable, the rank attributed by each algorithm and the dispersion of the rank attributed through the five algorithms. On the other hand, the gamma analysis technique was used to rank the most inter-groups discriminative variables^23,24^.

#### Models and classification

We used the Synthetic Minority Over-sampling Technique (SMOTE) to manage unbalanced groups’ size to avoid any bias in models’ learning and classification processes. To limit the number of candidate models and their complexity in order to be compatible with a physiological explainability and a bedside application, we generated only combinations of 1 to 5 variables among the 24 selected HRV variables, i. e. we generated $\sum_{i=1}^{i=5}{C}_{i}^{5}=31$ combinations among the $\sum_{i=1}^{i=5}{C}_{i}^{24}=55454$ possible combinations. From the 31 combinations of the most discriminative variables generated, 341 classifying models were generated applying on each combination 11 machine learning algorithms that were: logistic regression, decision tree, random forest, linear support vector machine (SVM), radial based function SVM, neuronal network, gradient boosting machine, stacked ensembles, extremely randomized trees, k nearest neighbor, and extreme gradient boosting. Database was randomly split into training (80% of sample size) and validation (20% of sample size) sets. Performances of the 341 classifying models for SSc subtypes classification purposes, i.e., dcSSc or lcSSc, were assessed according to 8 usual metrics that are: mean squared error, logloss, sensitivity, specificity, precision, accuracy, area under curve of the ROC-curves and F1-score. The cross validation was performed with k-fold = 5 and repeated 10 times, providing information on how well a classifier generalizes for test data and further classifications, in particular the expected error range of the classifier thanks to the cross-validation score.

### Ethical approval

The study was approved by the Ethical Committee of Marseille University Hospital and was conducted in accordance with the Declaration of Helsinki. Each subject provided written consent for data analysis. Agreement number is PADS19-352/WZTVF7.

## Results

### Population description

Between January 01, 2018, and December 31, 2021, 60 patients having SSc and assessed for dysautonomia were enrolled. 16 had dcSSc and 42 lcSSc clinical forms according to the internist experts. Two were excluded because of missing data. General characteristics of the studied population and each subgroup are summarized in Table 1. By definition and as expected, the two groups differed clinically mainly in the higher proportion of patients with severe symptoms in the dcSSc group compared with the lcSSc group, as observed through the higher Rodnan (t-statistic = 8.885, df = 20, p < 0.001) and Medsger (U-statistic = 626.5, p < 0.001) scores, and through the presence of sphincter atonia (χ^2^-statistic = 26.72, df = 2, p = 0.001) and nonspecific interstitial pneumonia (χ^2^-statistic = 44.86, df = 2, p = 0.031). Similarly, esophageal peristaltism dysfunction and diffuse parenchymal lung disease tended to be more frequent in the dcSSc group. As also expected, the two groups biologically differed mainly in the higher proportion of patients with anti-Scl-70 (χ^2^-statistic = 209.56, df = 2, p < 0.001) and anti-SSA (χ^2^-statistic = 20.5, df = 2, p = 0.001) antibodies in the dcSSc group compared with the lcSSc group that had higher proportion of patients with anti-centromere antibodies (χ^2^- = 14.05, df = 2, p = 0.001).

**Table 1:** General characteristics of the studied population.

	All SSc	dcSSc	lcSSc	p-value
**Anthropometry**				
Age, years	50.5 ± 13.8	47.4 ± 11.5	51.6 ± 14.5	0.108
Sex, F/M (%)	44/14 (76/24)	9/7 (16/12)	35/7 (60/12)	**0.068**
Weight, kg	65.8 ± 11.7	69.1 ± 12.3	64.0 ± 10.8	0.135
Height, m	1.66 ± 0.08	1.71 ± 0.10	1.65 ± 0.06	**0.044**
BMI, kg/m^2^	23.7 ± 3.4	23.6 ± 3.4	23.5 ± 3.2	0.905
**Clinical features and severity**				
Raynaud duration, years	12.9 ± 8.1	9.5 ± 4.1	14.2 ± 8.9	**0.01**
SSc duration, years	10.1 ± 6.3	8.6 ± 2.8	10.7 ± 7.1	**0.1**
Rodnan score, n.u.	6.8 ± 6.4	15.2 ± 4.8	3.6 ± 3.2	** < 0.001**
Medsger score, n.u.	4.9 ± 2.5	7.7 ± 1.6	3.7 ± 1.8	** < 0.001**
Pulmonary hypertension, y/n (%)	5/53 (9/91)	2/14 (4/24)	3/39 (5/67)	1
Oesophagal aperistaltism, y/n (%)	31/27 (53/47)	13/3 (22/5)	18/24 (31/42)	**0.064**
Sphincter atonia, y/n (%)	22/36 (28/72)	13/3 (22/5)	9/33 (16/57)	** < 0.001**
**Lung disease**				
Dyspnea, I/II/III/IV NYHA scale	33/23/2/0	7/7/2/0	26/16/0/0	0.176
Thorax TDM, y/n (%)	43/15 (74/26)	12/4 (21/7)	31/11 (53/19)	0.984
Diffuse parenchymal lung disease, y/n (%)	13/30 (30/70)	6/6 (14/14)	7/24 (16/56)	**0.076**
NSIP, y/n (%)	3/40 (7/93)	2/10 (5/23)	1/30 (2/70)	**0.031**
IPF, y/n (%)	3/40 (7/93)	1/11 (2/25)	2/29 (5/68)	0.965
Fibrosis, y/n (%)	10/33 (23/77)	4/8 (9/19)	6/25 (14/58)	0.472
Fibrosis > 20%, y/n (%)	8/35 (19/81)	3/9 (7/21)	5/26 (12/60)	0.705
**Biology**				
Anti-Scl-70, y/n (%)	31/26 (54/46)	15/1 (26/2)	16/25 (28/44)	** < 0.001**
Anti-centromere, y/n (%)	24/33 (42/58)	0/16 (0/28)	24/17 (42/30)	**0.001**
Anti-SSA, y/n (%)	8/49 (14/86)	8/8 (14/14)	0/41 (0/72)	**0.001**
Anti-SSB, y/n (%)	5/52 (9/91)	5/11 (9/19)	0/41 (0/72)	**0.054**
Anti-U1 RNP, y/n (%)	0/57 (0/100)	0/16 (0/28)	0/41 (0/72)	1
Anti-RNA polymerase III, y/n (%)	1/56 (2/98)	1/15 (2/26)	0/41 (0/72)	0.918
Anti-PM/Scl y/n (%)	0/57 (0/100)	0/16 (0/28)	0/41 (0/72)	1
Anti-fibrillarin, y/n (%)	1/56 (2/98)	0/16 (0/28)	1/40 (2/70)	0.988
**Treatments**				
Calcium channel blockers, y/n (%)	35/23 (60/40)	7/9 (12/16)	28/14 (48/24)	**0.011**
Diltiazem, y/n (%)	5/53 (9/91)	2/14 (3/24)	3/39 (5/68)	0.576
Non-diltiazem, y/n (%)	30/28 (52/48)	5/11 (9/19)	25/17 (43/29)	** < 0.001**
Proton-pump inhibitors, y/n (%)	10/48 (17/83)	4/12 (7/21)	6/36 (10/62)	0.276
Steroids, y/n (%)	2/56 (3/97)	1/15 (2/26)	1/42 (2/70)	0.585
l-thyroxin, y/n (%)	6/52 (10/90)	0/16 (0/28)	6/36 (10/62)	0.651
Chloroquin, y/n (%)	4/54 (7/93)	0/16 (0/28)	4/38 (7/65)	0.827
Sildenafil, y/n (%)	7/51 (12/88)	3/13 (5/22)	4/38 (7/66)	0.309
Mycophenolate, y/n (%)	12/46 (21/79)	9/7 (16/12)	3/39 (5/67)	** < 0.001**
Bosentan, y/n (%)	8/50 (14/86)	3/13 (5/22)	5/37 (9/64)	0.524
Other drugs, y/n (%)	14/44 (24/76)	3/13 (5/22)	11/31 (19/54)	0.466

Values are expressed as mean ± standard deviation or modality size (percentage of the whole population) for quantitative and categorical variables, respectively. The whole studied population (All SSc) are described in the first column and p-values of the comparison between diffuse (dcSSc) and located (lcSSc) cutaneous systemic sclerosis are reported. P < 0.05 was considered as significant and p < 0.1 (bold) was used as threshold for further analysis. BMI: Body mass index, SSc: Systemic sclerosis, NSIP: Nonspecific interstitial pneumonia, IPF: Idiopathic pulmonary fibrosis.

### Lung function

Respiratory parameters measured during lung function test and their interpretation as functional syndromes are summarized in Table 2. As expected, in terms of lung function, the two groups differed only in the higher proportion of patients with restrictive lung disease in the dcSSc group compared with the lcSSc group (χ^2^-statistic = 26.74, df = 2, p = 0.001).

**Table 2:** Lung function characteristics of the studied population.

	All SSc	dcSSc	lcSSc	p-value
**Lung function, values *(n*** **=** ***58)***				
TLC, L	4.87 ± 1.11	4.10 ± 0.89	5.15 ± 1.06	** < 0.001**
% of predicted	91 ± 21	71 ± 17	98 ± 17	** < 0.001**
FRC, L (%)	2.83 ± 0.60	2.53 ± 0.44	2.95 ± 0.62	**0.007**
% of predicted	99 ± 21	83 ± 14	105 ± 21	** < 0.001**
Slow VC, L (%)	3.03 ± 0.85	2,43 ± 0.63	3.26 ± 0.81	** < 0.001**
% of predicted	91 ± 27	65 ± 24	101 ± 20	** < 0.001**
Forced VC, L (%)	2.94 ± 0.87	2.31 ± 0.63	3.18 ± 0.83	** < 0.001**
% of predicted	91 ± 28	64 ± 26	101 ± 20	** < 0.001**
RV/TLC, no unit (%)	37.75 ± 7.88	40.63 ± 7.64	36.72 ± 7.79	**0.084**
% of predicted	105 ± 22	120 ± 26	100 ± 18	** < 0.001**
FEV_1_, L (%)	2.37 ± 0.67	1.90 ± 0.42	2.54 ± 0.67	** < 0.001**
% of predicted	87 ± 25	63 ± 21	96 ± 19	** < 0.001**
FEV_1_/forced VC, %, (%)	81.41 ± 7.70	83.75 ± 8.88	80.51 ± 7.12	0.163
% of predicted	103 ± 9	105 ± 10	102 ± 8	0.283
MMEF, L (%)	2.47 ± 1.06	2.12 ± 0.65	2.6 ± 1.17	0.443
% of predicted	73 ± 28	58 ± 16	79 ± 29	**0.001**
Raw cmH_2_0*s/L (%)	1.90 ± 0.74	1.91 ± 0.63	1.90 ± 0.78	0.711
% of predicted	62 ± 24	63 ± 21	62 ± 26	0.711
DLCO_sb_, mL/mmHg/min (%)	16.38 ± 5.19	14.48 ± 3.14	17.08 ± 5.63	**0.036**
% of predicted	65 ± 18	53 ± 12	69 ± 18	** < 0.001**
KCO_c_, mL/mmHg/min/L (%)	3.76 ± 0.78	3.99 ± 0.78	3.69 ± 0.77	0.225
% of predicted	80 ± 16	86 ± 16	78 ± 15	0.150
SpO_2_, %	99 ± 0.99	98 ± 1	99 ± 1	0.5
**Lung function, interpretation** ***(n*** **=** ***58)***				
Restrictive lung disease, y/n (%)	16/42 (28/72)	9/7 (16/12)	7/35 (12/60)	** < 0.001**
Lung distension, y/n (%)	1/57 (2/98)	0/16 (0/28)	1/41 (2/70)	0.988
Obstructive lung, y/n (%)	5/53 (9/91)	1/15 (2/26)	4/38 (7/65)	0.681
Lung diffusion impairement, y/n (%)	11/47 (19/81)	2/14 (3/24)	9/33 (16/57)	0.216

Values are expressed as mean ± standard deviation or modality size (percentage of the whole population) for quantitative and categorical variables, respectively. The whole studied population (All SSc) are described in the first column and p-values of the comparison between diffuse (dcSSc) and located (lcSSc) cutaneous systemic sclerosis are reported. P < 0.05 was considered as significant and p < 0.1 (bold) was used as threshold for further analysis. TLC, total lung capacity; FRC, functional residual capacity; slow VC, slow vital capacity; forced VC, forced vital capacity; RV/TLC, residual volume/total lung capacity ratio; FEV1, first 1-second forced expiratory volume; FEV1/forced VC, modified Tiffeneau-Pinelli index; MMEF, Maximal mid-expiratory flow; Raw, central airway resistance; DLCOsb, single-breath carbon monoxide diffusion capacity; KCOc, corrected carbon monoxide transfer coefficient; SpO_2_, oxyhemoglobin saturation rate estimated from pulse oximetry.

### Autonomic influences on sinus node activity

HRV indexes in decubitus then in orthostatism conditions as well as the stand-up induced HRV changes are summarized in Table 3. The two groups differed mainly in a higher HR in the dcSSc group compared with the lcSSc group (mean_HR, clinical form main effect, H-statistic = 11.13, p = 0.003), in decubitus and even more so in orthostatism position (mean_HR, interaction of main effects, H-statistic = 29.93, p < 0.001), HR (mean_HR) being inversely proportional to RRI duration (mean_RR). On the contrary, the differences in observed values for all other HRV markers, especially those derived from spectral and nonlinear analysis, appear to be less systematic. Except for ShanEn, which is similarly higher in the dcSSc group compared with the lcSSc group (ShanEn, clinical form main effect, F-statistic = 6.50, df = 1, p = 0.049) in both decubitus and orthostatic positions (no interaction effect), the other HRV markers differ from one group to another depending on the position considered. This differential effect of verticalization according to patients' group is highlighted through the interaction effect, as seen with LF_power, HF_power, or tot_power. Similarly, a higher length of the mean and the longest diagonal line of the recurrence plot was observed in the dcSSc group compared with the lcSSc group according to position (Lmax, interaction of main effects, H-statistic = 28.92, p < 0.001 and Lmean, interaction of main effects, H-statistic = 13.46, p = 0.011), Lmax being inversely linked to divergence (DIV). Finally, the two groups differed also in terms of HRV parameters changes induced by stand-up. Mainly, mean_HR increase was higher in dcSSc compared to lcSSc groups (t-statistic = 3.034, df = 24, p = 0.006) while all others time domain parameters were lowered during orthostatism and even more lowered in the dcSSc group than in the lcSSc group. Particularly, sd_HR was lowered in the dcSSc group while increased in the lcSSc group (t-statistic = − 3.329, df = 18, p = 0.004). Similarly, SD2 and CorDim were lowered in the dcSSc group while increased in the lcSSc group (t-statistic = − 3.586, df = 18, p = 0.002 and t-statistic = − 3.247, df = 20, p = 0.004, respectively).

**Table 3:** Heart rate variability of the studied population.

	Decubitus		Othostatism		Clinical form	Position	Interactions	Stand-up-induced changes		
	dcSSc	lcSSc	dcSSc	lcSSc	*p value*	*p value*	*p value*	DcSSc	LcSSc	*p value*
**Time domain**										
mean_RR (ms)	825 ± 120	894 ± 113	698 ± 117	795 ± 102	**0.002**^•^	< 0.001	1	− 127 ± 48	− 99 ± 52	0.072^•^
sd_RR (ms)	40 ± 22	33 ± 14	22 ± 11	34 ± 14	0.590	0.555	**0.035**^•^	− 18 ± 22	1 ± 9	**0.005**^•^
mean_HR (bpm)	74.0 ± 10.0	68.0 ± 9.0	88.0 ± 13.0	77.0 ± 11.0	**0.003**^•^	< 0.001	** < 0.001**	14.0 ± 6.0	9.0 ± 5.0	**0.006**^•^
sd_HR (bpm)	4.0 ± 2.0	3.0 ± 1.0	3.0 ± 1.0	3.0 ± 1.0	1	0.426	**0.047**^•^	− 1.0 ± 2.0	1.0 ± 1.0	**0.004**^•^
RMSSD (ms)	35 ± 29	28 ± 18	14 ± 13	22 ± 16	0.890	< 0.001	**0.002**^•^	− 21 ± 29	− 6 ± 10	0.052^•^
NN50 (count)	53.0 ± 61.0	30.0 ± 52.0	12.0 ± 30.0	22.0 ± 44.0	1	0.046	0.068	− 41.0 ± 60.0	− 8.0 ± 26.0	**0.048**^•^
pNN50 (%)	15.0 ± 17.0	9.0 ± 16.0	4.0 ± 9.0	6.0 ± 12.0	1	0.030	**0.048**	− 12.0 ± 16.0	− 3.0 ± 8.0	0.054^•^
**Frequency domain**										
LF_peak (Hz)	0.074 ± 0.022	0.076 ± 0.028	0.074 ± 0.025	0.073 ± 0.027	1	1	1	− 0.000 ± 0.00	− 0.003 ± 0.03	0.648
HF_peak (Hz)	0.264 ± 0.08	0.257 ± 0.07	0.24 ± 0.071	0.214 ± 0.069	1	0.008	**0.038**	− 0.024 ± 0.101	− 0.044 ± 0.105	0.393
LF_power (ms^2^)	750 ± 1574	369 ± 508	155 ± 218	434 ± 434	0.142	1	**0.013**^•^	− 595 ± 1602	65 ± 464	0.124
LF_power_prc (%)	31.0 ± 12.0	30.0 ± 13.0	27.0 ± 17.0	37.0 ± 16.0	0.461	1	0.302^•^	− 4.0 ± 16.0	7.0 ± 19.0	**0.040**^•^
LF_power_nu (n.u.)	54.0 ± 18.0	53.0 ± 18.0	71.0 ± 24.0	71.0 ± 19.0	1	< 0.001	** < 0.001**	17.0 ± 20.0	18.0 ± 23.0	0.485
HF_power (ms^2^)	712 ± 1385	326 ± 377	89 ± 152	192 ± 265	0.946	< 0.001	** < 0.001**^•^	− 623 ± 1391	− 134 ± 289	0.183
HF_power_prc (%)	27.0 ± 13.0	27.0 ± 17.0	13.0 ± 16.0	16.0 ± 15.0	1	< 0.001	** < 0.001**	− 13.0 ± 13.0	− 11.0 ± 16.0	0.686
HF_power_nu (n.u.)	46.0 ± 18.0	46.0 ± 18.0	29.0 ± 24.0	28.0 ± 19.0	1	< 0.001	** < 0.001**	− 17.0 ± 20.0	− 18.0 ± 23.0	0.496
LF_HF_power	1.54 ± 1.08	1.61 ± 1.55	4.72 ± 4.05	4.59 ± 4.6	1	< 0.001	** < 0.001**	3.18 ± 3.67	2.98 ± 4.9	0.866
tot_power (ms^2^)	2055 ± 3373	1135 ± 1004	559 ± 567	1218 ± 1081	0.657	0.502	0.092^•^	− 1496 ± 3474	83 ± 982	0.092^•^
**Nonlinear domain**										
SD1 (ms)	25 ± 20	20 ± 13	10 ± 9	15 ± 11	0.890	< 0.001	**0.002**^•^	− 15 ± 20	− 4 ± 7	0.052^•^
SD2 (ms)	50 ± 24	42 ± 17	29 ± 13	45 ± 19	0.898	0.559	**0.034**^•^	− 21 ± 26	3 ± 13	**0.002**^•^
ApEn	1.139 ± 0.067	1.112 ± 0.11	1.006 ± 0.176	1.071 ± 0.105	1	0.003	**0.015**	− 0.133 ± 0.169	− 0.041 ± 0.138	**0.034**^•^
SampEn	1.561 ± 0.275	1.58 ± 0.268	1.171 ± 0.359	1.298 ± 0.25	0.832	< 0.001	1	− 0.391 ± 0.415	− 0.281 ± 0.282	0.257
alpha1	1.045 ± 0.288	1.047 ± 0.248	1.261 ± 0.355	1.308 ± 0.313	1	< 0.001	** < 0.001**	0.216 ± 0.319	0.261 ± 0.285	0.636
alpha2	0.964 ± 0.161	0.928 ± 0.177	1.12 ± 0.206	0.979 ± 0.221	0.133	0.12	0.801^•^	0.156 ± 0.215	0.051 ± 0.239	0.054^•^
CorDim	2.082 ± 1.703	1.358 ± 1.367	0.759 ± 1.163	1.449 ± 1.367	1	0.745	0.115^•^	− 1.323 ± 1.619	0.091 ± 1.039	**0.004**^•^
Lmin (beats)	2.0 ± 0.0	2.0 ± 0.0	2.0 ± 0.0	2.0 ± 0.0	1	1	1	0.0 ± 0.0	0.0 ± 0.0	1.0
Lmax (beats)	235.0 ± 126.0	159.0 ± 75.0	320.0 ± 145.0	285.0 ± 115.0	0.194	< 0.001	** < 0.001**^•^	85.0 ± 115.0	126.0 ± 108.0	0.168
Lmean (beats)	12.5 ± 3.7	11.0 ± 4.0	15.0 ± 4.7	12.6 ± 3.5	0.090	0.009	**0.011**^•^	2.5 ± 6.0	1.6 ± 3.5	0.496
DIV	0.006 ± 0.003	0.008 ± 0.006	0.004 ± 0.003	0.005 ± 0.005	0.194	< 0.001	** < 0.001**^•^	− 0.002 ± 0.003	− 0.003 ± 0.005	0.163
REC (%)	0.368 ± 0.118	0.315 ± 0.097	0.399 ± 0.097	0.373 ± 0.084	0.198	0.021	1	0.032 ± 0.139	0.058 ± 0.092	0.686
DET (%)	98.0 ± 1.5	97.6 ± 1.3	98.9 ± 1	98.7 ± 1.2	0.200	< 0.001	** < 0.001**	0.9 ± 1.3	1.1 ± 1.1	0.374
ShanEn	3.262 ± 0.312	3.111 ± 0.302	3.475 ± 0.357	3.293 ± 0.31	**0.049**^•^	0.006	1	0.212 ± 0.44	0.183 ± 0.265	0.776

Values are expressed as mean ± standard deviation. Diffuse (dcSSc) and located (lcSSc) cutaneous systemic sclerosis are described for decubitus and orthostatism and adjusted (Bonferroni’s correction) p-values of the clinical form and position main effects as their interaction are reported. Stand-up-induced changes of each group are also reported. P < 0.05 was considered as significant (bold). Unadjusted p-values<0.1 from univariate analysis (^•^ mark) was used as threshold for further analysis. Time domain variables are: mean_RR, mean RR intervals duration; sd_RR, standard deviation of RR intervals duration; mean_HR, mean heart rate; sd_HR, standard deviation of heart rate; RMSSD, root mean square of successive differences of RR intervals duration; NN50, count of successive RR intervals duration differing from more than 50 ms; pNN50, percentage of RR intervals duration differing from more than 50 ms. Frequency domain variables are: LF_peak and HF_peak, low frequency (0.04–0.15 Hz) and high frequency (0.15–0.40  Hz) having the highest power peaks, respectively; LF_power, low frequency power; LF_power_prc, percentage of low frequency power; LF_power_nu, normalized low frequency power; HF_power, high frequency power; HF_power_prc, percentage of high frequency power; HF_power_nu, normalized high frequency power; LF_HF_power, low frequency/high frequency power ratio; tot_power, total spectral power. Nonlinear variables are: SD1, minor axis of the fitted ellipse on RR intervals Poincaré plot; SD2, major axis of the fitted ellipse on RR intervals Poincaré plot; ApEn, approximate entropy; SamEn, sample entropy; alpha1, first (short-term) alpha coefficient of detrended fluctuation analysis of RR intervals time series; alpha2, second (long-term) alpha coefficient of detrended fluctuation analysis of RR intervals time series; CorDim, correlation dimension; Lmin, minimal line length; Lmax, maximal line length; Lmean, mean line length; DIC, divergence; REC, recurrence rate; DET, determinism; ShanEn, Shannon entropy.

### Features importance

**Figure 1:**
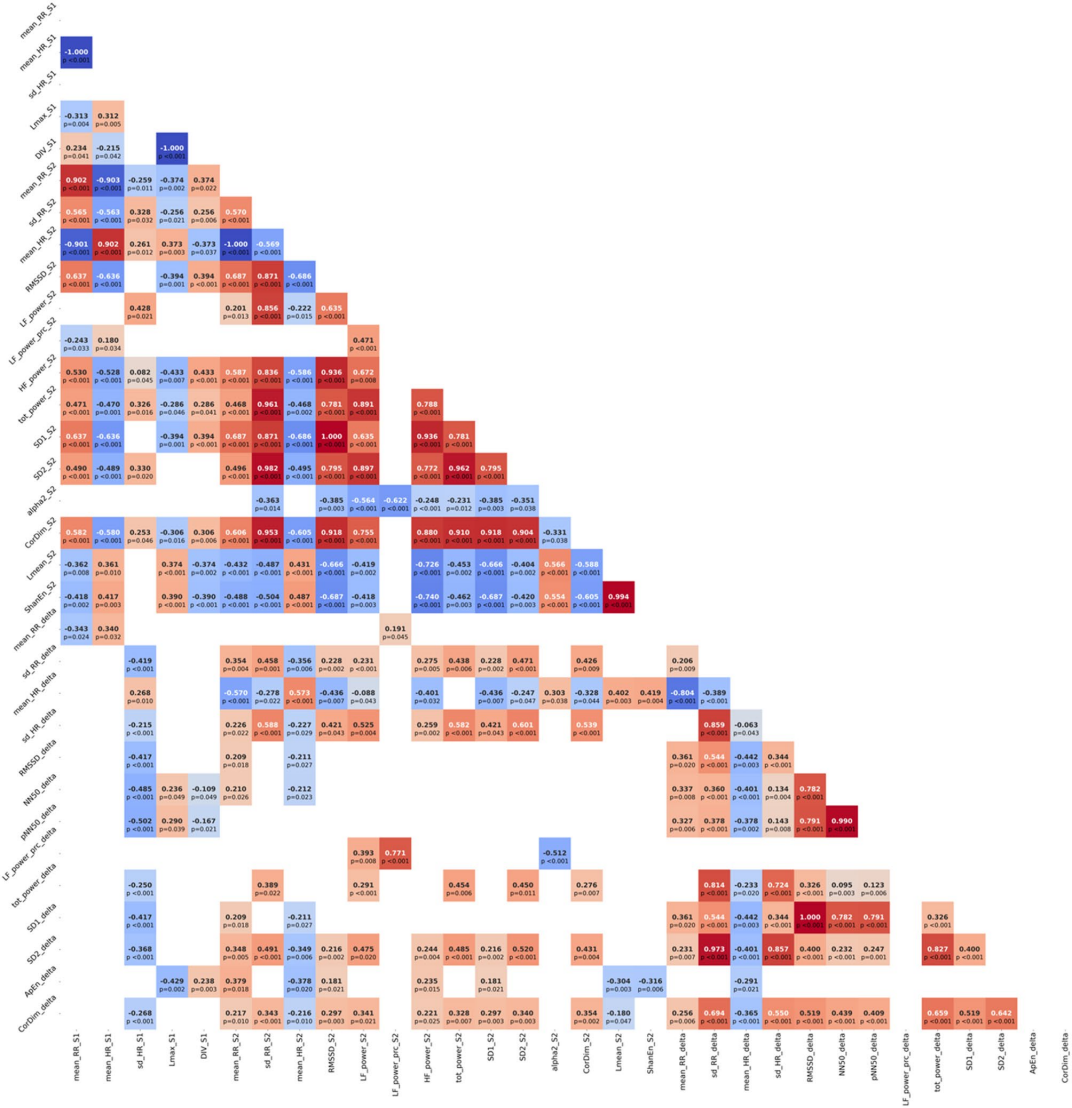
Correlations between heart rate variability metrics. The HRV metrics that were significantly different (p < 0.05) in the dcSSc and lcSSc subgroups were tested for correlation and are represented in the correlation matrix. Correlations between HRV metrics from time-, frequency-, and nonlinear- domains measured in supine (_S1) as orthostatism (_S2) conditions and HRV metrics stand-up induced changes (_delta) are reported. R^2^ is color-coded from -1 (dark red) to + 1 (dark blue) when correlation was significant (p < 0.05).

**Figure 2:**
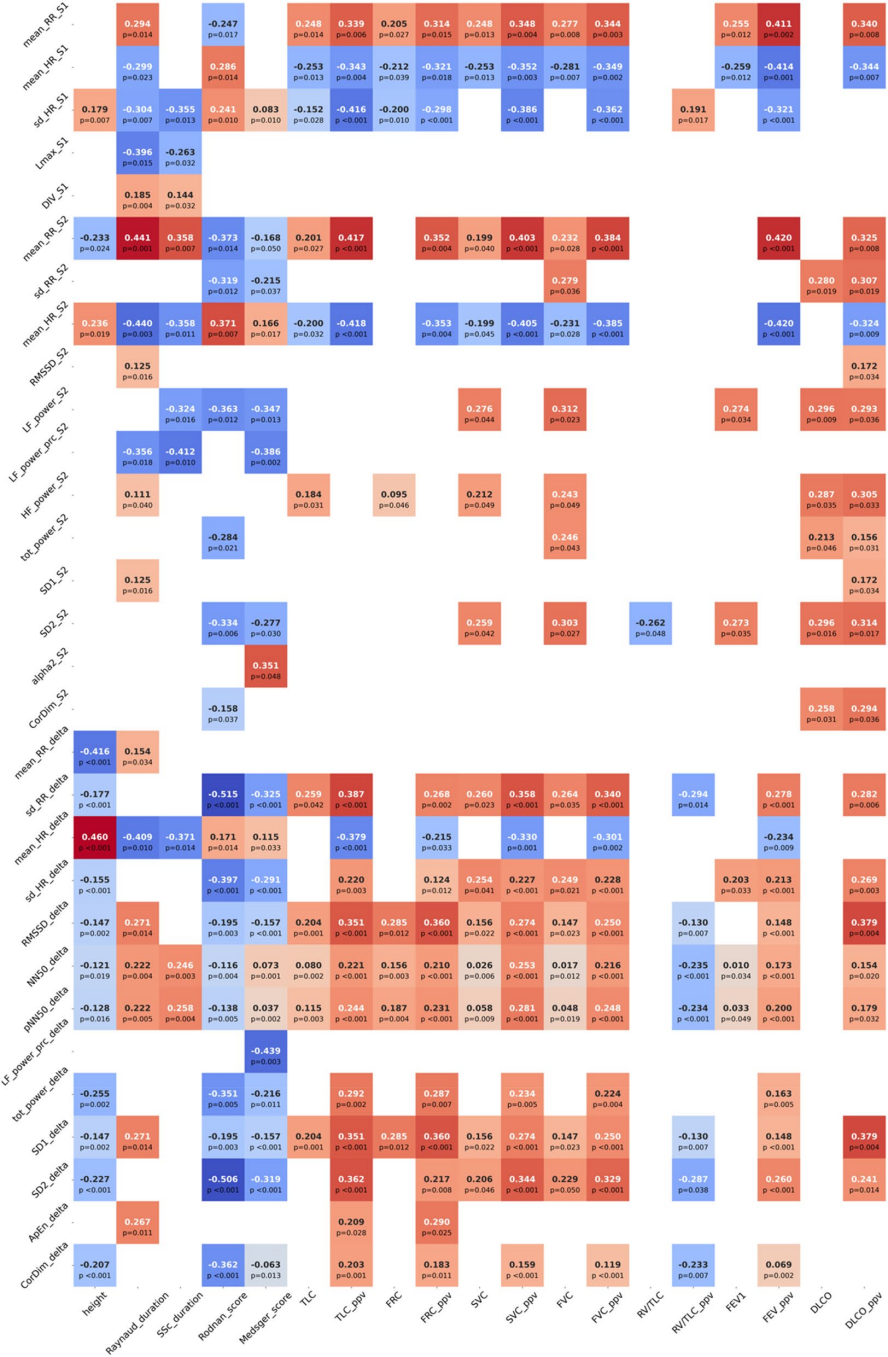
Correlations between clinical features, heart rate variability and lung function metrics. Correlations between HRV metrics from time-, frequency-, and nonlinear- domains measured in supine (_S1) as orthostatism (_S2) conditions and HRV metrics stand-up induced changes (_delta), and clinical features as well lung function metrics are reported. R^2^ is color-coded from -1 (dark red) to + 1 (dark blue) when correlation was significant (p < 0.05).

**Figure 3:**
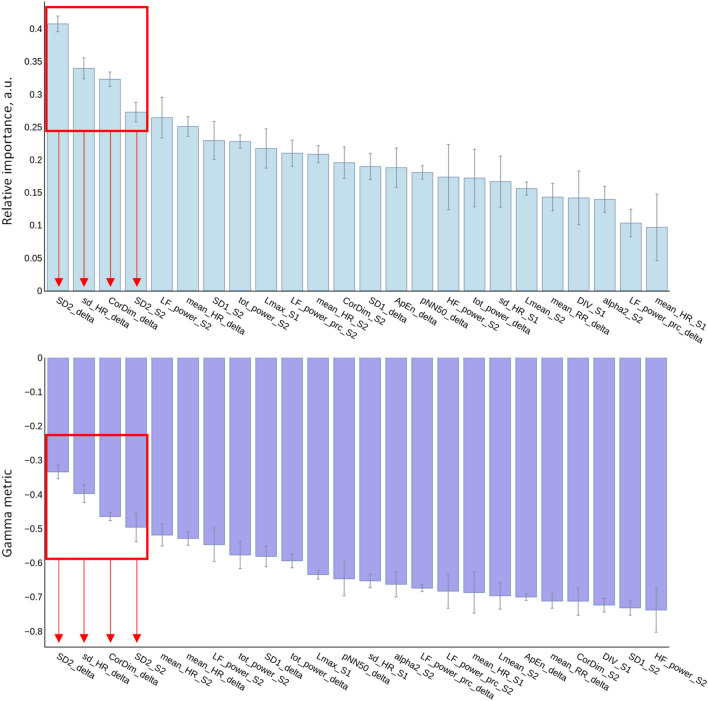
Features importance. The features importance of heart rate variability metrics is represented according to two different features importance ranking techniques: relative importance (top histograms), and gamma-metric (bottom histograms). Features importance of HRV metrics from time-, frequency-, and nonlinear- domains measured in supine (_S1) as orthostatism (_S2) conditions and HRV metrics stand-up induced changes (_delta) was assessed. The four most important features (red arrows) were: SD2_delta, stand-up induced change of the major axis length of the fitted ellipse on RR intervals Poincaré plot; sd_HR_delta, stand-up induced change of the heart rate standard deviation; CorDim_delta, stand-up induced change of the correlation dimension; SD2_S2, major axis length of the fitted ellipse on RR intervals Poincaré plot in orthostatism condition.

Collinearity and correlations between all variables that have been significantly different in the dcSSc group compared to the lcSSc group with a univariate approach are summarized in Figs. 1 and 2. We can note that many HRV indexes are positively or negatively correlated, and mostly of interest, some HRV parameters are correlated to clinical and respiratory quantitative parameters. Particularly, first, mean_HR_S2, SD2_delta, sd_HR_delta, SD2_delta and CorDim_delta are correlated with Rodnan and Medsger scores and with most of the respiratory function parameters, and second, most of the HRV parameters changes (delta) are correlated with clinical scores and lung function parameters. A synthetic view of features importance to describe data variance is proposed in Fig. 3. Features are ranked according two different metrics, the relative importance of the feature (explained data variance) and the gamma-metric (discriminant power). The first four most efficient features identically identified are SD2_delta, sd_HR_delta, CorDim_delta, and SD2_S2. The fifth important feature is either LF_power_S2 either mean_HR_S2 according to the used metric. As fifth feature we chose mean_HR_S2 because of a more physiological and measuring robustness.

### Models’ performances

Table 4 summarizes the performances of the classifying models generated including 1 to 5 selected variables. Concerning sensibility and specificity we can note that models range from 0.87 to 1 and from 0.25 to 0.99 respectively. The most efficient variable taken alone and combining the best sensibility and specificity combination is CorDim with 1 and 0.99 values respectively. But on the other hand, precision, accuracy, and F1-score are the lowest of all tested models (0.749, 0.771, and 0.823 respectively). Besides, it should be noted that the proposed models, except for the one including only CorDim, appear to have a rather average sensitivity ranging from 0.25 to 0.81. Moreover and interestingly, we can note that F1-score that is an integrative metrics (harmonic mean of precision and accuracy) is not a linear function of the number of included variables. F1-score ranges from 0.823 for the best 1-variable model to a maximum of 0.947 for the 4-variables best model while 0.946 is reached by the 3-variables best model. If specificity and/or precision is an objective we can note that best models are two 3-variables and two 4-variables models including SD2_delta, SD2_S2, sd_HR_delta, and CorDim_delta.

**Table 4:** Performances of classifying models.

Model variables	Classifier algorithm	MSE	LogLoss	Sensibility	Specificity	Precision	Accuracy	AUC	F1-score
**1 variable**									
mean_HR_S2	Neuronal network	0.17	0.578	0.96	0.583	0.858	0.869	0.762	0.897
SD2_delta	Neuronal network	0.157	0.433	0.95	0.5	0.841	0.847	0.875	0.88
SD2_S2	Neuronal network	0.162	0.492	0.92	0.563	0.837	0.829	0.782	0.852
sd_HR_delta	Neuronal network	0.182	1.192	1	0.25	0.72	0.762	0.759	0.828
CorDim_delta	Stacked Ensemble	0.188	0.53	1	1	0.729	0.749	0.771	0.823
**2 variables**									
SD2_delta, mean_HR_S2	GBM	0.117	0.341	1	0.583	0.858	0.891	0.9	0.919
CorDim_delta, mean_HR_S2	Neuronal network	0.154	0.465	1	0.583	0.858	0.891	0.848	0.919
SD2_delta, CorDim_delta	GBM	0.155	0.468	0.92	0.708	0.916	0.891	0.88	0.903
sd_HR_delta, mean_HR_S2	GBM	0.137	0.423	1	0.521	0.825	0.869	0.888	0.901
SD2_S2, mean_HR_S2	Neuronal network	0.159	0.453	1	0.604	0.833	0.871	0.88	0.901
sd_HR_delta, CorDim_delta	Neuronal network	0.186	0.546	0.91	0.625	0.882	0.869	0.88	0.888
sd_HR_delta, SD2_S2	Neuronal network	0.155	0.503	0.87	0.688	0.922	0.869	0.83	0.878
SD2_delta, SD2_S2	Neuronal network	0.141	0.438	1	0.396	0.783	0.824	0.859	0.873
CorDim_delta, SD2_S2	Neuronal network	0.199	0.616	0.95	0.375	0.806	0.802	0.802	0.854
SD2_delta, sd_HR_delta	Neuronal network	0.162	0.515	1	0.313	0.738	0.784	0.811	0.841
**3 variables**									
SD2_delta, SD2_S2, mean_HR_S2	GBM	0.101	0.311	1	0.771	0.902	0.936	0.943	0.946
SD2_delta, CorDim_delta, mean_HR_S2	Neuronal network	0.126	0.434	0.96	0.688	0.916	0.911	0.903	0.935
CorDim_delta, SD2_S2, mean_HR_S2	Neuronal network	0.133	0.54	1	0.563	0.881	0.889	0.826	0.929
sd_HR_delta, CorDim_delta, mean_HR_S2	Neuronal network	0.123	0.465	0.95	0.625	0.898	0.891	0.868	0.913
SD2_delta, sd_HR_delta, SD2_S2	Neuronal network	0.165	0.515	1	0.5	0.847	0.867	0.863	0.911
SD2_delta, CorDim_delta, SD2_S2	Neuronal network	0.147	0.454	1	0.5	0.847	0.867	0.887	0.911
sd_HR_delta, SD2_S2, mean_HR_S2	Neuronal network	0.208	0.77	1	0.521	0.825	0.869	0.875	0.901
SD2_delta, sd_HR_delta, mean_HR_S2	GBM	0.112	0.331	0.95	0.563	0.881	0.869	0.878	0.9
SD2_delta, sd_HR_delta, CorDim_delta	Neuronal network	0.162	0.541	1	0.375	0.806	0.822	0.833	0.884
sd_HR_delta, CorDim_delta, SD2_S2	Neuronal network	0.212	0.748	1	0.417	0.781	0.827	0.834	0.871
**4 variables**									
SD2_delta, CorDim_delta, SD2_S2, mean_HR_S2	GBM	0.076	0.245	0.96	0.813	0.935	0.933	0.917	0.947
sd_HR_delta, CorDim_delta, SD2_S2, mean_HR_S2	Neuronal network	0.23	0.83	1	0.625	0.898	0.911	0.903	0.942
SD2_delta, sd_HR_delta, SD2_S2, mean_HR_S2	GBM	0.097	0.302	0.95	0.813	0.942	0.936	0.935	0.94
SD2_delta, sd_HR_delta, CorDim_delta, mean_HR_S2	GBM	0.115	0.348	0.971	0.771	0.885	0.891	0.913	0.916
SD2_delta, sd_HR_delta, CorDim_delta, SD2_S2	Neuronal network	0.213	0.708	1	0.5	0.847	0.867	0.863	0.911
**5 variables**									
SD2_delta, sd_HR_delta, CorDim_delta, SD2_S2, mean_HR_S2	GBM	0.092	0.279	1	0.708	0.878	0.913	0.909	0.931

SSc subtypes classifying models including 1 to 5 most informative and discriminant HRV variables are presented. Variables used are: SD2_delta, stand-up induced change of the major axis length of the fitted ellipse on RR intervals Poincaré plot; sd_HR_delta, stand-up induced change of the heart rate standard deviation; CorDim_delta, stand-up induced change of the correlation dimension; SD2_S2, major axis length of the fitted ellipse on RR intervals Poincaré plot in orthostatism condition; mean_HR_S2, mean heart rate in orthostatism condition. Classifier algorithms used were neuronal networks; stacked ensembles; and GBM, gradient boosting machines. Models’ performances were assessed through MSE, mean squared error; LogLoss, logarithmic loss; Sensibility; Specificity; Precision; Accuracy; AUC, area under curve; and F1-score.

### Secondary outcomes

The link between HRV metrics and SSc skin extent appears through some significant regressions, the most significant simple linear regressions being Rodnan score = -165.063 × SD2_delta + 6.261 (R^2^ = 0.268, F-statistic: 19.80, df = 1, p < 0.001) and Medsger score = − 0.062 × LF_power_prc_delta + 5.143 (R^2^ = 0.210, F-statistic: 14.36, df = 1, p < 0.001). The most significant multiple linear regressions provide Rodnan score estimation from mean_RR_S2, mean_HR_S2, ApEn_S2, alpha1_S2, Lmean_S2, DIV_S2, DET_S2, and ShanEn_S2 (R^2^ = 0.842, F-statistic: 4.453, df = 30, p < 0.001) and Medsger score estimation from alpha1_S2, CorDim_S2, and Lmean_S2 (R^2^ = 0.756, F-statistic: 2.393, df = 30, p = 0.015). Similarly, the link between HRV metrics and lung function appears through some significant regressions, the most significant simple logistic regression expressing the presence of a restrictive syndrome as a function of mean_HR_S2 (pseudo R^2^ = 0.277, likelihood statistic: − 32.542, df = 1, p < 0.001) and the most significant multiple logistic regression expressing the presence of a restrictive syndrome as a function of SD2_S2 and sd_RR_S2 (pseudo R^2^ = 0.55, likelihood statistic: − 32.542, df = 8, p < 0.001).

## Discussion

### Major findings

We aimed to provide classifying models of SSc subtypes, i.e., models that distinguish dcSSc from lcSSc clinical forms, from HRV linear and nonlinear metrics, and easy to compute at the patient’s bedside. As expected, at rest in decubitus condition, only HR was clearly different in SSc subgroups, HR being higher in dcSSc. Transition from decubitus to orthostatism increased HR in all SSc patients. HR reached higher magnitude in dcSSc and was associated with a lower overall variability (tot_power). Stand-up-induced HRV changes revealed differential effects of orthostatism in SSc subtypes, effects that were mainly higher HR increase associated with (1) loss of time-domain variability and (2) loss of nonlinear proprieties of HRV (sd_RR, sd_HR, SD1 and SD2, ApEn, CorDim, and recurrence plot quantifying indexes) in dcSSc compared to lcSSc patients. Accordingly, efficient classifying models of SSc subtypes have been provided using from 1 to 4 HRV metrics as input variables reaching F1-score of overall performance up to almost 0.95. To resume, it’s possible to distinguish dcSSc from lcSSc clinical forms at the patient’s bedside with a simple digital ECG analysis.

### Autonomic influences on sinus node activity

Time domain and frequency domain HRV metrics have been used to investigate the cardiovascular consequences of SSc since 1994^2^. On one hand, SSc is known to alter autonomic nervous system and its cardiovascular component^25^ and on the other hand HRV has been considered to inform on autonomic nervous system modulation of sinus node rhythmic activity^13^. High frequency (around 0.25 Hz) being related to respiration via the parasympathetics and low frequency (around 0.1 Hz) to a sympathetic Eigen-oscillation of the baroreflex to blood pressure and heart rate system^26^. Typically, SSc is described as increasing resting HR through a predominant sympathetic activity and vagal withdrawal compared to healthy controls^9,27–29^ and as blunting cardiovascular response to orthostatism^9,29^. The HR and HRV we observed in dcSSc and lcSSc subtypes at rest and during orthostatism does not evidence a so clear phenotype-dependent sympathetic activation neither parasympathetic withdrawal. In decubitus position, when comparing dcSSc and lcSSc groups using multiple univariate unpaired t-tests as well as ANOVA, we found no evidence for any difference in autonomic modulation of sinus activity, as commonly accepted^13^. For 5-min RR intervals time series this usually includes some HRV spectral indexes that are LF_power (ms^2^), LF_power_prc (%), LF_power_nu (n.u.), HF_power (ms^2^), HF_power_prc (%), HF_power_nu (n.u.) and LF_HF_power. On the contrary, in dcSSc compared to lcSSc, we found that orthostatism was paradoxically associated with significantly lower low frequency spectral power (LF_power as explicated by the main effects interaction) while high frequency spectral power (HF_power as explicated by the main effects interaction) tends to be lower. Orthostatism is known to physiologically induce a temporary adaptative response increasing sympathetic and decreasing parasympathetic activities. Oppositely to previous descriptions^9,29^, our data do not reveal any major alteration in the sympathovagal response, as expressed by LF_prc, LF_nu, HF_prc, HF_nu and LF_HF ratio, to orthostatism. These normal responses are explicitely evident in Table 3 of the results. LF_power_nu and even more so HF_power, HF_power_prc, HF_power_nu, as well as LF_HF_power, are significantly modified during orthostatism compared to decubitus (position effect, p < 0.001). No clinical form effect (group effect) was observed but only main effects interactions (main effects interactions, p < 0.001). The effect of orthostatism was different according to clinical form. Yet, indexes from RRI time series spectral analysis are known to be markers of the autonomic nervous system influences on the sinus node rhythmic activity. Particularly LF and HF are thought to reflect sympathovagal balance as well as sympathetic and parasympathetic sinus influences, respectively, when expressed in normalized units. According to first HRV guidelines^13^, one could interpret our results as a sympathovagal balance functionality. On the contrary, there are at least two factors that should prompt us to approach the interpretation more judiciously. Firstly, a recent and highly regarded review^30^ emphasizes that scientific evidence in the literature supports a predominantly parasympathetic-determined Heart Rate Variability (HRV) when assessed through spectral analysis indexes. This implies that, based on the results of HFnu and LFnu, as well as others spectral analysis outcomes, it appears that parasympathetic-determined HRV does not play a major role in partitioning the clinical forms of SSc patients. Secondly, the mathematical constructs of normalized HRV spectral metrics can lead to a confusing interpretation, depending on whether LF and HF relatively vary with LFnu = LF/(TP − VLF) ≈ LF/(LF + HF) and HFnu = HF/(TP − VLF) ≈ HF/(LF + HF). According to these knowledges and our observations, it is difficult to interpret our results as a simple and unique parasympathetic blunted response (withdrawal) to transition from decubitus to orthostatic conditions. It seems that in orthostatism, sympathetic (LF_power (ms2), LF_power_prc (%)) modulation of sinus activity is lower in dcSSc compared to lcSSc that is in favor of an unpaired adaptation to orthostatism all the more so as it should increase. As LFnu = LF/(TP − VLF), this impairment seems to be supported by modifications impacting LF, TP, but also VLF that is said to reflect mid- and long- term renin-angiotensin system, blood pressure regulation, thermoregulation, and others long-term humoral and hormonal activities^13^. And second, Poincaré plot short term variability index SD1 was not significantly different between dcSSc and lcSSc groups (no clinical form effect) but orthostatism had differential effects according the groups (main effects interactions, p < 0.002). SD1, that is the standard deviation of the plot data on the minor axis is mathematically equal to root mean square of successive differences of RRI duration described the instantaneous beat-to-beat variability that is neurally supported by the only vagal nerve^31^. Oppositely, Poincaré plot long term variability index SD2 that is said to reflect sympathetic and baroreflex HR modulation^32,33^ was significantly different between dcSSc and lcSSc groups during orthostatism but decubitus (main effects interactions, p < 0.034). All these elements converge with the exclusion of a simple vagal-mediated impairment differential main effect between lcSSc and dcSSc, but mostly toward a baroreflex loop complex impairment. Probably for this reason, metrics of HRV linear dynamics lacked to clearly distinguish dcSSc and lcSSc subtypes.

### HR nonlinear dynamics and system failure

Three metrics quantifying the nonlinear properties of HR dynamics have been found to be more deeply impacted in dcSSc vs. lcSSc groups by orthostatic stress according to stand-up-induced changes results: SD2, ApEn, and CorDim. SD2 that is the long/major axe of the elliptic fit of Poincaré plot is also said to reflect nonlinear long-term RR modulation^31,32^. ApEn reflects the complex structure of a time series and the complex behavior of the system generating the time series, i.e., the system complexity and its components interactions^34–38^. CorDim quantifies the time self-similarity of a signal, i.e., of the RRI time series. Thus, CorDim can be considered as an estimation of the lower threshold of the number of degrees of freedom for the underlying system generating the observed data and is typically used as an index of the overall complexity of the system dynamics estimated from a time series^21^. In healthy people, stand-up induces a loss of HRV because of the arterial baroreflex, an adaptative autonomic nervous system response associating sympathetic activation and parasympathetic withdrawal^39^. But the complexity of a physiological system is increased when its components are interacting to adequate physiological response^34^. Taken together, the combined stand-up-induced changes of these three metrics that explore complementary nonlinear properties highlight that the cardiovascular homeostatic system is impaired in dcSSc but not in lcSSc. Stand-up stimulus should increase nonlinearity of HR dynamics, which is a wellness index of physiological systems. But in our study, stand-up decreased HR nonlinearity and complexity, which is an impairment and failure marker of dynamic systems including physiological complex systems^34^. Accordingly, our results converge to highlight that orthostatism stress lowers dramatically physiological flexibility (adaptability) in dcSSc patients compared to lcSSc. DcSSc are less adaptative to orthostatism than lcSSc. These results seem to be in accordance with previous data on baroreflex impairment and orthostatism intolerance in SSc patients. Normal vasoconstrictive response to an increase in venous transmural pressure is almost abolished in tissues with sclerodermic changes, probably due to sympathetic neuropathy^40^. Besides, advanced and fibrotic forms of SSc are associated with a blunted orthostatic stress response^9^. Finally, orthostatic stress intolerance is significantly associated with microvascular damage as assessed by videocapillaroscopy^41^. Moreover, in addition to the above-mentioned pathophysiological elements, we cannot exclude but have even to retain the potential role of the pulmonary abnormalities mainly observed in the dcSSc patients and characterizing restrictive lung disease in the loss of HRV nonlinear properties. It is well-known that lung expansion magnitude determines respiratory sinus arrythmia that is a major component of heart rate variability through vagal activity^42–44^. Now, we have precisely documented that dcSSc exhibited more frequently restrictive syndrome than lcSSc and that the presence of a restrictive syndrome was correlated with HRV differences at rest, during orthostatism, and changes induced by stand-up test.

### SSc subtypes and SSc phenotyping

SSc phenotyping into dcSSc and lcSSc remains a daily challenge for practitioners as clinical form phenotype is a determinant of prognosis and care strategy of the disease^1^. In 2019, Sobanski et al. used 24 clinical and serological variables to cluster 6927 SSc patients in an elegant data analysis study^45^. The first 2 clusters overlapped the usual dcSSc and lcSSc subtypes but authors showed that these 2 clusters may not capture the complete heterogeneity of the disease, several other clusters being associated with differential survival rates. They concluded that organ damage should be taken into consideration. Among other criteria that could help SSc phenotyping, HRV has been previously studied^3–6,9^. Nevertheless, no major difference had been previously robustly documented and no operational tool has been developed and tested for a real-life application. Through this study we provided SSc subtypes clinical forms classifying models based on HRV parameters usable at the patient’s bedside. Several models are provided, more or less complex, but reasonably easy to compute for a daily use, with different performances allowing the clinician to choose the most appropriate for his medical purpose. The easiest models are that provided by our secondary outcomes. HRV can model SSc skin extent trough linear regressions and model respiratory function, mainly the presence of a restrictive syndrome, through logistic regressions. The first being part of the very definition of SSc clinical forms, the second being a highly known pathological status associated with dcSSc. Nevertheless, the efficient models (R^2^ > 0.5) are quickly complex in terms of number of included variables and interpretability. As shown in Table 2, most of the lung function test variables were significatively different in lcSSc and dcSSc. But most of these variables are correlated and mostly were highly predictable by linear regression models (results not shown). Because of this data high collinearity, probably because of a causal link that could be a limited stretch reflex from the restrictive lungs (neither demonstrated nor documented in this work), we had to provide a specific methodology to extract the most informative variables that we performed through features engineering step. To our knowledge, we propose for the first time a whole functional test of cardiovascular function, usable at the patient’s bedside that includes: (1) a 15-min (10 min decubitus, stan-up, 5 min orthostatism) 1-lead numerical ECG recording, (2) the estimation of 1 to 5 HRV metrics easy to compute, and (3) a simple classifying model using machine learning approaches. Locally, we have chosen the GBM 4-variables classifying model including SD2_delta, CorDim_delta, SD2_S2, and mean_HR_S2 HRV indexes as it has the best specificity and F1-score compromise. Nevertheless, the 1-variable model including the only CorDim has the best sensibility–specificity combination while its F1-score is the lowest (0.82) of the tested models that is, in data science an honorable performance. Finally, the choice of the model has to be driven by the underlying medical question and expectation.

### Limits

#### Physiological interpretation

A first potential limitation of this study is that we did not directly assess sympathetic and parasympathetic tones by neurography. Physiological and physio-pathological interpretation of the models is then not obvious. We used indirect indexes (spectral powers) from spectral analysis of RRI time series that are known to capture only linear properties of HR dynamics. Nevertheless, in stationary conditions, LF_power and HF_power, expressed in ms^2^ as well as in normalized units, are largely thought to reflect sympathetic and parasympathetic modulatory effects on sinus node activity^13^. As previously discussed, compiling knowledge on effects of sympathetic and vagal activity on HRV suggests that the HRV power spectrum, including its low frequency component, is mainly determined by the parasympathetic system^30^. Thus, LF-power and HF_power are said to be of limited interest in some physiological conditions, especially during interacting processes that, by definition, lead to nonlinear behavior^46,47^. In relation to the complexity of the sinus node activity modulation system, predominantly nonlinear behavior must be assumed, explored, and interpreted.

#### Length of time series

A second potential limitation of this study is the number of points constituting the analyzed time series. On one hand, the length of RRI time series used to perform linear time- and frequency-domains HRV analysis is usually set to 5 min^13^. The value of the scaling exponent is mostly defined by the dynamics of the short-time variability. On the other hand, and oppositely, exploring nonlinear dynamics as the dimensionality of the space spanned by the data, particularly when using CorDim, requires long time series to be reliably computed^48^. Time series are supposed to include 10^CorDim^ data points, i.e., around 10,000 data points should be used. Using only and approximately 400 data points per time series, we can’t consider that CorDim we computed represent robustly and reliably the whole concept and the underlying properties defined by the correlation dimension. But decidedly, the computation we performed (that of CorDim, i.e., the correlation sum) on 400 data points time series led to a metrics that was statistically characterized as sensitive, reliable, stable and discriminant marker. Accordingly, we then highlight that the interpretation of the time behavior of the RRI time series and HR dynamics should be made with caution: The CorDim changes we measured are not necessarily the results of the changes in the scaling behavior of the heart rate dynamics. Specific studies should test this hypothesis.

#### Design of the study

A third limitation of the study could be the necessary precaution to take before generalizing these results. Most of the previous studies were designed as sex and age matched groups analysis. On the contrary, we have taken 60 consecutive patients and did not perform specific matching. As shown in Table 1, no age or sex difference appeared but height was significantly different in dcSSc and lcSSc. Three points needs to be addressed to consolidate conclusions: even if our sample size was sufficient for data analysis and to provide, *in fine*, a validated set of models, sample size has not been calculated on all variables of interest of the study that has not been designed accordingly; no matching has been made on any variable; and no early-stage disease group has been identified since all patient had already their diagnosis. Ormea et al.^25^ showed that autonomic degeneration was independent of tissue and vascular alterations, as severe nerve damage was already present in apparently healthy skin. He suggested that ANS modification takes place before vascular damage and tissue fibrosis occur. Moreover, Ormea stated that ANS dysfunction is a main factor in the development of the disease. Accordingly, further studies on a larger dataset are needed to confirm and generalize our first results, particularly with the aim to use the classifying models as a predicting tool when used at the early-stage of the disease.

## Conclusion, contribution and prospects

In conclusion, this study provides high performance classifying models able to distinguish dcSSc from lcSSc based on only 1 to 5 HRV indexes used as markers of autonomic integrated influences on cardiac activity. The best classifying model are a gradient boosting machine and a neural network reaching a 0.94 F_1_-score that include 4 HRV indexes capturing nonlinear properties of HR dynamics. Even if the division of SSc in dcSSc and lcSSc gives mainly a clue of clinical and prognostic stratification, knowing as soon as possible the clinical subtype may impact patient’s care. Easy to compute at the patient’s bedside, these classifying models need external validation step and have to be tested to predict subtype and clinical form at the early-stage of the disease before an intensive daily medical use.

## Data Availability

The data that support the findings of this study are available from Assistance Publique–Hôpitaux de Marseille, Marseille, France, but restrictions apply to the availability of these data and so are not publicly available. Data are however available from the authors upon reasonable request and with permission of Assistance Publique–Hôpitaux de Marseille, Marseille, France.
